# The complete mitochondrial genome of the common vole, *Microtus arvalis* (Rodentia: Arvicolinae)

**DOI:** 10.1080/23802359.2018.1457994

**Published:** 2018-04-04

**Authors:** Remco Folkertsma, Michael V. Westbury, Jana A. Eccard, Michael Hofreiter

**Affiliations:** a Department of Mathematics and Natural Science, Evolutionary Adaptive Genomics, Institute for Biochemistry and Biology, University of Potsdam, Potsdam, Germany;; b Department of Mathematics and Natural Sciences, Animal Ecology, Institute for Biochemistry and Biology, University of Potsdam, Potsdam, Germany;; c Natural History Museum of Denmark, University of Copenhagen, Copenhagen, Denmark

**Keywords:** *Microtus arvalis*, Arvicolinae, mitochondrial genome, common vole, phylogeny

## Abstract

The common vole, *Microtus arvalis* belongs to the genus Microtus in the subfamily Arvicolinae. In this study, the complete mitochondrial genome of *M. arvalis* was recovered using shotgun sequencing and an iterative mapping approach using three related species. Phylogenetic analyses using the sequence of 21 arvicoline species place the common vole as a sister species to the East European vole (*Microtus levis*), but as opposed to previous results we find no support for the recognition of the genus Neodon within the subfamily Arvicolinae, as this is, as well as the genus Lasiopodomys, found within the Microtus genus.

The genus *Microtus* is one of the most diverse genera in the subfamily Arvicolinae. Consisting of about 70 different species it is one of the fastest radiating mammalian genera (Nowak 1999). The common vole (*Microtus arvalis*) is a small rodent widely distributed throughout Eurasia, ranging from the Atlantic coast in France to Central Russia. They experience a range of different climatic conditions from sea level to high altitude in the Alps (Fischer et al. 2011) and occupy a variety of different habitats such as farmland and grassland, making it a popular study species in ecological and evolutionary research. The species shows high levels of between population genetic differentiation (Heckel et al. 2005) and strong genetic clustering among populations on small scales (Schweizer et al. 2007). Although previous studies have used partial mitochondrial DNA sequences to show the presence of five main evolutionary lineages and to resolve their phylogenetic positioning within the genus *Microtus* (Fink et al. 2004), the complete mitochondrial genome of *M. arvalis* has not yet been published.

The male *M. arvalis* used in this study was sampled in 2015 outside the town of Lochow, Germany (52.690640°N, 12.455182°E) under permits from Landesumweltamt Brandenburg (RW-7.1 24.01.01.10). A voucher specimen was deposited at the University of Potsdam, Potsdam, Germany. DNA was extracted using the Qiagen DNeasy kit, built into an Illumina sequencing library and sequenced using an Illumina NextSeq 500. We assembled the full mitochondrial genome using an iterative mapping approach (Hahn et al. 2013) with three independent runs utilizing available Arvicolinae as reference bait sequences. Resultant sequences were aligned using Mafft v7.271 (Katoh and Standley 2013) and a final consensus sequence was built using Genious v9.0.5 (Kearse et al. 2012). We obtained a circular sequence, 16,286 bp in length (GenBank Accession No. MG948434) which was annotated using MITOS (Bernt et al. 2013). Finally, a phylogenetic analysis was performed on an alignment of our consensus sequence, all available complete mitochondrial arvicoline sequences and *Cricetulus griseus*. We produced a maximum-likelihood phylogenetic tree of all 13 protein-coding genes and tRNA genes, with an appropriate partitioning scheme and GTR + G as the substitution model as determined by PartitionFinder (Lanfear et al. 2012), with 1000 bootstrap replicates using RAxML-HPC2 on XSEDE v8.2.10 (Stamatakis 2014) on the Cipres server (Miller et al. 2010) (Figure 1).

**Figure 1.**
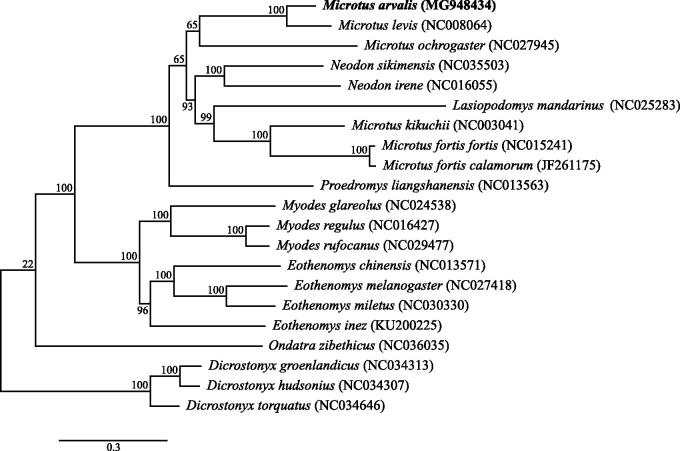
Maximum-likelihood tree of the subfamily Arvicolinae based on the sequences of 13 protein-coding genes and the tRNA genes of the mitochondrial genome using *Cricetulus griseus* as an outgroup (not shown here for graphical reasons). Bootstrap support values are shown at the branch nodes.

Phylogenetic analysis shows a well-supported sister-clade relationship between Arvicolini and Myodini. Furthermore, in concordance with other studies using mitochondrial or nuclear loci (Jaarola et al. 2004; Fink et al. 2010), we also find *M. arvalis* to be a sister species of *Microtus levis*. However, in contrast to Galewski et al. (2006) who used the mitochondrial *Cytb* and nuclear *Ghr* genes, we found no support for the recognition of the genus *Neodon*, as this is found within the genus *Microtus*, as is the genus *Lasiopodomys*. Thus, mitogenomic analysis suggests that these genera should be subsumed within the *Microtus* genus. We hope publication of the mitochondrial genome of *M. arvalis* will help to understand the phylogenetic relationship within the Arvicolinae and the genus *Microtus*.

## References

[CIT0001] Bernt M, Donath A, Jühling F, Externbrink F, Florentz C, Fritzsch G, Pütz J, Middendorf M, Stadler PF. 2013 MITOS: improved de novo metazoan mitochondrial genome annotation. Mol Phylogenet Evol. 69:313–319. 22982435 10.1016/j.ympev.2012.08.023

[CIT0002] Fink S, Excoffier L, Heckel G. 2004 Mitochondrial gene diversity in the common vole *Microtus arvalis* shaped by historical divergence and local adaptations. Mol Ecol. 13:3501–3514. 15488007 10.1111/j.1365-294X.2004.02351.x

[CIT0003] Fink S, Fischer MC, Excoffier L, Heckel G. 2010 Genomic scans support repetitive continental colonization events during the rapid radiation of voles (Rodentia: *Microtus*): the utility of AFLPs versus mitochondrial and nuclear sequence markers. Syst Biol. 59:548–572. 20834011 10.1093/sysbio/syq042

[CIT0004] Fischer MC, Foll M, Excoffier L, Heckel G. 2011 Enhanced AFLP genome scans detect local adaptation in high-altitude populations of a small rodent (*Microtus arvalis*). Mol Ecol. 20:1450–1462. 21352386 10.1111/j.1365-294X.2011.05015.x

[CIT0005] Galewski T, Tilak M, Sanchez S, Chevret P, Paradis E, Douzery EJ. 2006 The evolutionary radiation of Arvicolinae rodents (voles and lemmings): relative contribution of nuclear and mitochondrial DNA phylogenies. BMC Evol Biol. 6:80. 17029633 10.1186/1471-2148-6-80 PMC1618403

[CIT0100] Hahn C, Bachmann L, Chevreux B 2013 Reconstructing mitochondrial genomes directly from genomic next-generation sequencing reads - a baiting and iterative mapping approach. Nucleic Acids Res. 41:e129. 23661685 10.1093/nar/gkt371 PMC3711436

[CIT0006] Heckel G, Burri R, Fink S, Desmet J-F, Excoffier L. 2005 Genetic structure and colonization processes in European populations of the common vole, *Microtus arvalis*. Evolution (NY). 59:2231–2242. 16405166

[CIT0007] Jaarola M, Martínková N, Gündüz İ, Brunhoff C, Zima J, Nadachowski A, Amori G, Bulatova NS, Chondropoulos B, Fraguedakis-Tsolis S, et al 2004 Molecular phylogeny of the speciose vole genus *Microtus* (Arvicolinae, Rodentia) inferred from mitochondrial DNA sequences. Mol Phylogenet Evol. 33:647–663. 15522793 10.1016/j.ympev.2004.07.015

[CIT0008] Katoh K, Standley DM. 2013 MAFFT multiple sequence alignment software version 7: improvements in performance and usability. Mol Biol Evol. 30:772–780. 23329690 10.1093/molbev/mst010 PMC3603318

[CIT0009] Kearse M, Moir R, Wilson A, Stones-Havas S, Cheung M, Sturrock S, Buxton S, Cooper A, Markowitz S, Duran C, et al 2012 Geneious Basic: an integrated and extendable desktop software platform for the organization and analysis of sequence data. Bioinformatics. 28:1647–1649. 22543367 10.1093/bioinformatics/bts199 PMC3371832

[CIT0010] Lanfear R, Calcott B, Ho SYW, Guindon S. 2012 PartitionFinder: combined selection of partitioning schemes and substitution models for phylogenetic analyses. Mol Biol Evol. 29:1695–1701. 22319168 10.1093/molbev/mss020

[CIT0011] Miller MA, Pfeiffer W, Schwartz T. 2010. Creating the CIPRES science gateway for inference of large phylogenetic trees. In: 2010 Gatew Comput Environ Work. New Orleans (LA): IEEE. p. 1–8.

[CIT0012] Nowak R. 1999 Walker’s mammals of the world. Baltimore (MD): Johns Hopkins University Press.

[CIT0013] Schweizer M, Excoffier L, Heckel G. 2007 Fine-scale genetic structure and dispersal in the common vole (*Microtus arvalis*). Mol Ecol. 16:2463–2473. 17561906 10.1111/j.1365-294X.2007.03284.x

[CIT0014] Stamatakis A. 2014 RAxML version 8: a tool for phylogenetic analysis and post-analysis of large phylogenies. Bioinformatics. 30:1312–1313. 24451623 10.1093/bioinformatics/btu033 PMC3998144

